# Research Progress on Graphite-Derived Materials for Electrocatalysis in Energy Conversion and Storage

**DOI:** 10.3390/molecules27248644

**Published:** 2022-12-07

**Authors:** Shuaijie He, Mingjie Wu, Song Li, Zhiyi Jiang, Hanlie Hong, Sylvain G. Cloutier, Huaming Yang, Sasha Omanovic, Shuhui Sun, Gaixia Zhang

**Affiliations:** 1 Engineering Research Center of Nano-Geomaterials of Ministry of Education, China University of Geosciences, Wuhan 430074, China; 2 Faculty of Materials Science and Chemistry, China University of Geosciences, Wuhan 430074, China; 3 School of Earth Sciences, China University of Geosciences, Wuhan 430074, China; 4 Department of Chemical Engineering, McGill University, 3610 University Street, Montreal, QC H3A 0C5, Canada; 5 Institut National de la Recherche Scientifique (INRS), Centre Énergie Matériaux Télécommunications, Varennes, QC J3X 1P7, Canada; 6 Department of Electrical Engineering, École de Technologie Supérieure (ÉTS), Montreal, QC H3C 1K3, Canada; 7 Hunan Key Laboratory of Mineral Materials and Application, School of Minerals Processing and Bioengineering, Central South University, Changsha 410083, China

**Keywords:** graphite-derived materials, fullerenes, carbon nanotubes, graphene, electrocatalysis

## Abstract

High-performance electrocatalysts are critical to support emerging electrochemical energy storage and conversion technologies. Graphite-derived materials, including fullerenes, carbon nanotubes, and graphene, have been recognized as promising electrocatalysts and electrocatalyst supports for the oxygen reduction reaction (ORR), oxygen evolution reaction (OER), hydrogen evolution reaction (HER), and carbon dioxide reduction reaction (CO_2_RR). Effective modification/functionalization of graphite-derived materials can promote higher electrocatalytic activity, stability, and durability. In this review, the mechanisms and evaluation parameters for the above-outlined electrochemical reactions are introduced first. Then, we emphasize the preparation methods for graphite-derived materials and modification strategies. We further highlight the importance of the structural changes of modified graphite-derived materials on electrocatalytic activity and stability. Finally, future directions and perspectives towards new and better graphite-derived materials are presented.

## 1. Introduction

**Figure 1:**
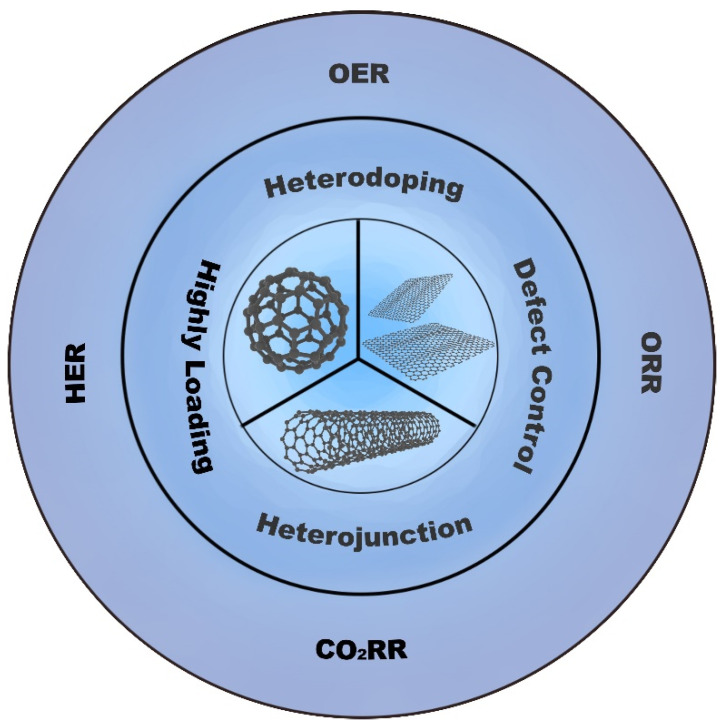
Graphite-derived materials and modification approaches used to develop advanced electrocatalysts for electrochemical energy storage and conversion systems based on the four redox reactions depicted in the figure.

The sustainable development of green and clean energy systems is one of the most complex problems facing human society. The vigorous development of electrochemical energy storage and conversion systems, such as new metal-air cells, fuel cells, water splitting, and carbon dioxide reduction, has pointed out a new direction for solving world energy problems. However, the slow reaction in core electrochemical reactions, including the oxygen reduction reaction (ORR), oxygen evolution reaction (OER), hydrogen evolution reaction (HER), and carbon dioxide reduction reaction (CO_2_RR), have become the bottleneck restricting the development of new energy technologies. Noble metal materials like platinum (Pt), iridium (Ir), and ruthenium (Ru) have been found to be the most efficient and selective electrocatalysts [1,2,3]. However, the limited reserves and the poor selectivity of these noble metals gravely impede practical applications of the technologies [4,5]. Carbon materials are promising substitutes for noble-metal-based electrocatalysts due to their abundant resources and easy modification. Among them, graphite-derived materials (fullerene, carbon nanotubes, and graphene) have attracted much attention owing to magnificent characteristics, such as high surface area, electron carrier mobility, and excellent catalytic activity [6,7,8]. This review intends to summarize recent progress in emerging electrocatalysts based on graphite-derived materials (Figure 1). Moreover, we discuss various means to boost electrocatalytic performance based on summarizing the structures and properties of different graphite-derived materials. Besides, the challenges and outlooks in this field are also presented to clarify the current situation of the reconstruction strategy of graphite-derived materials and the rational designs of high-performance electrocatalysts.

## 2. Overview of Electrocatalysis

Electrocatalysis with high catalytic activity and superior durability is required to achieve high power density and stability for electrochemical energy storage and conversion devices. Nevertheless, the core electrochemical reactions, such as ORR, OER, HER, and CO_2_RR, have high overpotential, and slow electron transfer dynamics, which has dramatically hindered the development of electrocatalysis [9,10,11]. Therefore, the use of highly active and selective catalysts to overcome the kinetics barriers related to the multi-step electron transfer process characterizing these reactions plays a pivotal role in electrocatalysis [12,13].

**Figure 2:**
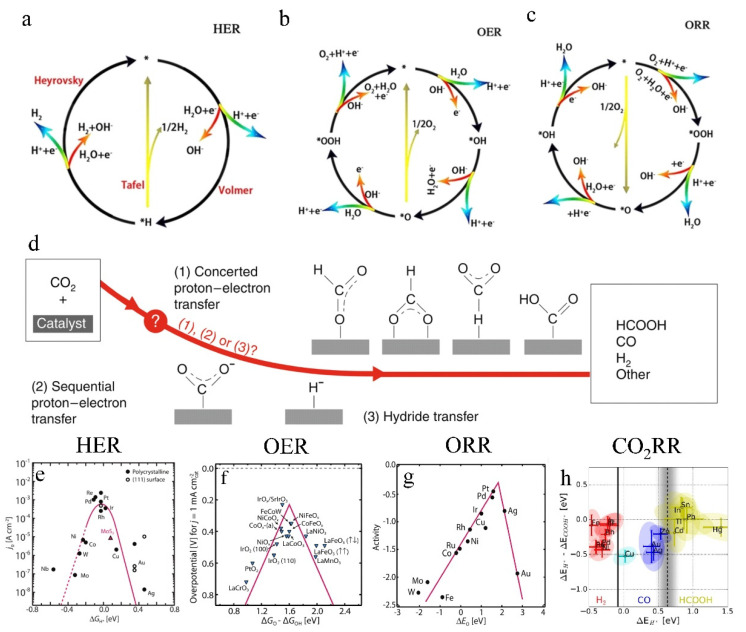
(**a**) HER mechanism in acid (blue line) and alkaline (red line) electrolytes. (**b**) OER mechanism in acid (blue line) and alkaline (red line) electrolytes. (**c**) ORR mechanism in acid (blue line) and alkaline (red line) electrolytes. Reproduced with permission [8]. Copyright 2020, Elsevier Ltd, Amsterdam, Netherlands. (**d**) CO_2_RR mechanism. Reproduced with permission [24]. Copyright 2019, Nature Publishing Group. (**e**) HER volcano plot for metals and MoS_2_. (**f**) OER volcano plot for metal oxides. (**g**) ORR volcano plot for metals. Reproduced with permission [17]. Copyright 2017, American Association for the Advancement of Science. (**h**) CO_2_ reduction metal classification. Reproduced with permission [25]. Copyright 2017, Wiley-VCH Verlag GmbH & Co. KGaA, Weinheim, Germany.

Electrochemical water splitting is a green, environmentally friendly, and efficient way to produce hydrogen, which involves two reactions: OER and HER. Among them, HER involves a double-electron transfer process, including the adsorption of water molecules or protons on the active site on the electrocatalyst surface (the Volmer step) and the desorption of hydrogen molecules from the cathode through the Tafel or Heyrovsky pathway (Figure 2a) [8,14,15]. Compared with HER, the OER involves a complex four-electron-proton transfer process and multiple reaction intermediates (Figure 2b), resulting in slower kinetics and higher overpotentials. OER restricts water-splitting development and is a significant constraint for new energy technologies such as regenerative fuel cells and rechargeable metal-air batteries [8,16,17,18,19].

The ORR reaction processes can generally be divided into two types: the four-electron (4-e^−^) reduction pathway, which directly transports oxygen to produce water; and the two-electron (2-e^−^) reduction pathway, which involves the conversion of oxygen to hydrogen peroxide and then to water (Figure 2c) [8]. The slow reaction kinetics of the ORR hinder the development of fuel cells and metal-air batteries [8,20,21,22]. Pt plays a significant role in ORR catalysis [23].

The CO_2_RR reaction mainly consists of three steps: chemisorption of CO_2_ from the electrolyte to the catalyst surface, electron or proton transfer to break C-O bonds and form C-H bonds, and product desorption from the catalyst surface(Figure 2d) [24,25]. This reaction provides a clean, sustainable route for producing high-value-added fuels and chemical precursors [26]. However, factors such as the chemical inertness of CO_2_, the reaction competition between HER and CO_2_RR, and the complex intermediates generated by the multiproton and electron reaction process lead to sluggish CO_2_RR kinetics [24,27,28]. Meanwhile, Cu-based materials are considered the only heterogeneous catalysts that promote the formation of various byproducts (e.g., hydrocarbon products and oxygenates) from the CO_2_RR [29].

Efficient catalysts can effectively reduce reaction barriers, promote the conversion of reaction intermediates, and accelerate reaction kinetics [30]. Therefore, developing highly active catalysts has become the top priority in developing the above-mentioned electrochemistry-based energy conversion and storage systems. The electrocatalytic activity largely depends on the binding energy between the reaction intermediates and the catalyst surface. Empirically, the bond strength between the catalyst and reaction intermediates is neither too strong nor too weak, which is shown by the volcano trends (Figure 2e–h) that can be used to evaluate the intrinsic activity of the electrocatalyst [17,25].

To comprehensively evaluate the catalytic activity of catalysts in the ORR, OER, HER, and CO_2_RR, the following standardized parameters will be considered further in the text: including overpotential (ηX, X represents current density), onset overpotential (E_onset_), half-wave potential (E_1/2_), electrochemical impedance spectroscopy (EIS), electrochemical active surface area (ECSA), turnover frequency (TOF), faradaic efficiency (FE), current density, limiting current density, Tafel slope, and stability.

## 3. Research Status on Graphite-Derived Materials

The graphite-derived materials such as fullerenes, carbon nanotubes, and graphene are widely used in the preparation of electrochemical catalysts due to their high specific surface area, environmental friendliness, excellent electrical properties, and the easiness of their surface functionalization.

Graphite, which is listed as a strategic mineral for crucial development and protection by some countries [31], is widely used in electrocatalysis [32], environmental protection [33], energy storage [34], refractories [31], thermal management [35], and many other industries. It has become an indispensable non-metallic material for many new strategic sectors. Fullerenes, carbon nanotubes, graphene, and other graphite-derived materials further broaden the application space of graphite in electrocatalysis.

Since Kroto discovered fullerene (C_60_) for the first time in the experiment of laser irradiation and evaporation of graphite, he then successively discovered fullerene molecules such as C_70_, C_80_, and C_90_. C_60_, which have high stability and ideal spherical structure. These fullerene molecules are considered the most representative zero-dimensional carbon material [36]. Due to the highly degenerate molecular energy level and small energy range, C_60_ has a high electronic affinity and solid chemical activity. It is often used as an electron acceptor to construct composite functional materials [37]. Currently, the main preparation methods of fullerenes include the laser, arc, and chemical synthesis methods [38].

**Figure 3:**
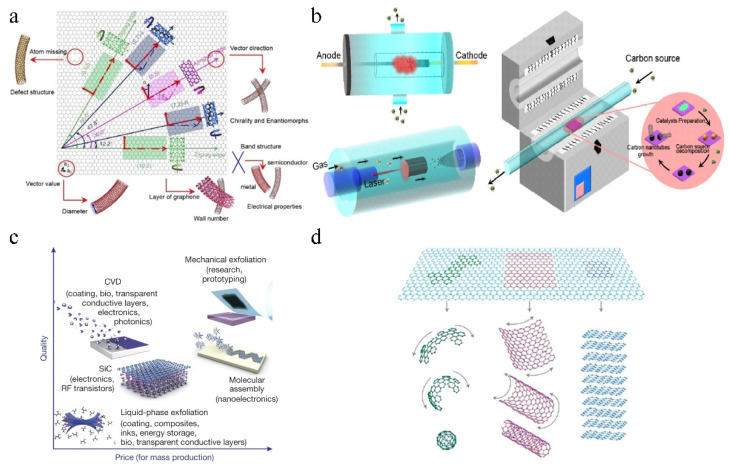
(**a**) Classification of carbon nanotubes (CNTs). (**b**) Techniques employed for the synthesis of CNT. Reproduced with permission [42]. Copyright 2020, China Academic Journal Electronic Publishing House. (**c**) Methods of mas-production of graphene. Reproduced with permission [50]. Copyright 2012, Nature Publishing Group. (**d**) Schematic diagram of graphene as a building unit to form fullerene, carbon nanotubes, and graphite. Reproduced with permission [52]. Copyright 2007, Nature Publishing Group.

Iijima of NEC in Japan accidentally discovered carbon nanotubes while preparing carbon fibers [39]. Carbon nanotubes can be divided into armchair-type carbon nanotubes, sawtooth-type carbon nanotubes, and chiral-type carbon nanotubes according to different crimping directions of graphene [40,41]. Based on the different layers of graphene, carbon nanotubes can be named single-walled carbon nanotubes (SWCNTs), double-walled carbon nanotubes (DWCNTs), and multi-walled carbon nanotubes (MWCNTs) (Figure 3a). Currently, arc-discharge, laser ablation, and chemical vapor deposition (CVD) are commonly used to prepare carbon nanotubes (Figure 3b) [42]. The tubes as carriers are characterized by high electrical conductivity, large specific surface area, and adjustable surface [43], which can greatly improve the conductivity of supported catalysts [44,45].

Graphene was successfully obtained by the research group of Professor Geim through mechanical stripping [46]. Graphene, as a two-dimensional carbon material, is composed of carbon atoms with sp^2^ hybrid orbital in a hexagonal honeycomb arrangement, which can be divided into a single-layer, double-layer, few-layers (3–10 layers) and multi-layer graphene (more than 10 layers, and less than 10 nm). Because of its good mechanical properties, extremely high carriers (electrons and holes) migration speed, superior electrical conductivity, and huge specific surface area [47,48], the star material has aroused great attention in many fields [49]. The existing preparation methods for graphene mainly include mechanical liquid phase, electrochemical, (CVD), and oxidation-reduction methods, which allow the synthesis of a wide range of graphene in terms of size, quality, and price for any particular application (Figure 3c) [50,51]. The zero-dimensional (0D) fullerenes, one-dimensional (1D) carbon nanotubes, and two-dimensional (2D) graphene constitute a family of graphite-derived materials (Figure 3d). Graphite is composed of multilayer graphene with weak van der Waals force. Carbon nanotubes can be regarded as graphene sheets rolled, which is attributed to the sp^2^ hybridization of carbon atoms and part of the sp^3^ hybridization. Fullerenes are made by bending graphene into balls. Therefore, graphene is the basic structural unit of various carbon sp^2^ hybrids materials such as fullerenes, carbon nanotubes, and graphite. Furthermore, graphite-derived materials that can be compounded with each other can improve the specific surface area, electron mobility, and energy band structure, thereby effectively improving the electrocatalytic activity [52].

## 4. Research Status on Fullerenes in Electrocatalysis

### 4.1. Doped Fullerene

**Figure 4:**
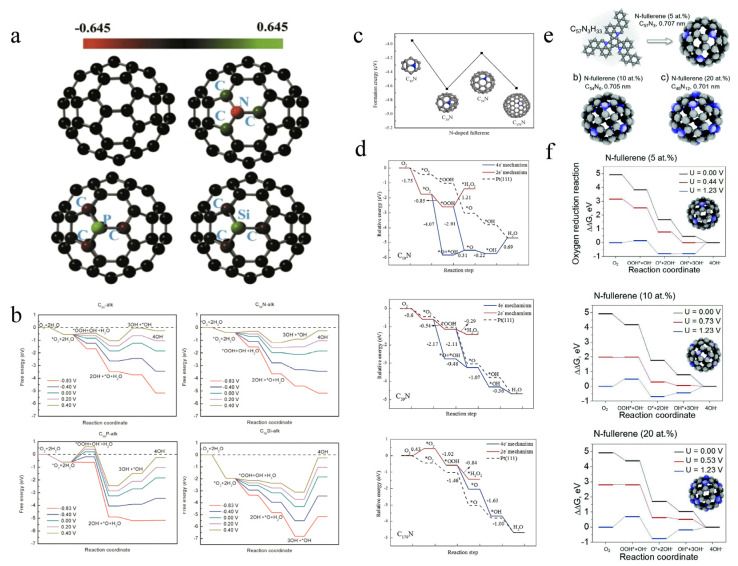
(**a**) Mulliken charge distribution on C_60_, C_59_N, C_59_P, C_59_Si. (**b**) Free-energy diagrams for the reduction of O_2_ at different electrode potentials, U, in alkaline medium on C_60_, C_59_N, C_59_P, C_59_Si. Reproduced with permission [55]. Copyright 2017, Elsevier Ltd. (**c**) Calculated formation energies of N-doped fullerenes. (**d**) Relative energy profiles of the possible ORR pathways. Reproduced with permission [56]. Copyright 2017, Elsevier Ltd. (**e**) N-Fullerene of the 5 at% N aromatic precursor with C_57_N_3_H_33_ molecules, and azafullerenes doped with N-doping levels of 10 and 20 at%, respectively. (**f**) Gibbs free energy diagrams of ORR in alkaline media with N-doping levels of 5, 10, and 20 at%. Used with permission [57]. Copyright 2017, The Royal Society of Chemistry.

Heteroatom doping can change the intrinsic electronic properties, atomic spin, charge density, energy band structure, and electronic state of carbon materials. Consequently, it can improve the electrocatalytic activity of carbon materials by introducing defects, holes, and more catalytically active sites [53,54]. The doping modification of C_60_ by heteroatoms (N, B, S, P, Si) has received extensive attention. Wang et al. [55] studied the influence of N, P, and Si doping on the catalytic activity of C_60_ in ORR by density functional theory (DFT). They found that heteroatom doping induces charge redistribution (Figure 4a). Besides, the catalysts’ free energy curve has been proved to be an efficient method to estimate the ORR catalytic performance. It shows that C_59_N and C_59_Si were the best and worst ORR catalysts, respectively (Figure 4b). Meanwhile, C_60_ with the high curvature and pentagonal defect has a high ORR catalytic activity. Chen et al. [56] paid attention to the ORR mechanism and catalytic performance of pure fullerenes and N-doped fullerenes in combination with DFT. They investigated the size effect of pure doped fullerenes on the ORR activity. The results reveal that the smallest (C_20_ and C_19_N) and the largest (C_180_ and C_179_N) fullerenes enable strong adsorption of the ORR species. In contrast, C_39_N with the reduced energy of the rate-determining step manifests a high ORR activity. Furthermore, the catalytic ORR pathway on C_39_N was predicted: O_2_→*O_2_→*O + *OH→*O + H_2_O→*OH + H_2_O→2H_2_O (Figure 4c,d). Seung Hyo Noh et al. [57] discussed the effect of nitrogen doping content on the OER and ORR catalytic activities of nitrogen-doped fullerenes. Combined with DFT calculations, the experiment showed that nitrogen-doped fullerenes with a 10% doping content had a higher bifunctional catalytic activity (Figure 4e,f). In conclusion, strategies such as introducing defects and doping can be used to develop efficient fullerene-based metal-free electrocatalysts for electrochemical energy storage and conversion systems.

### 4.2. Fullerene-Based Composites

#### 4.2.1. Metals and Metal Oxides

**Figure 5:**
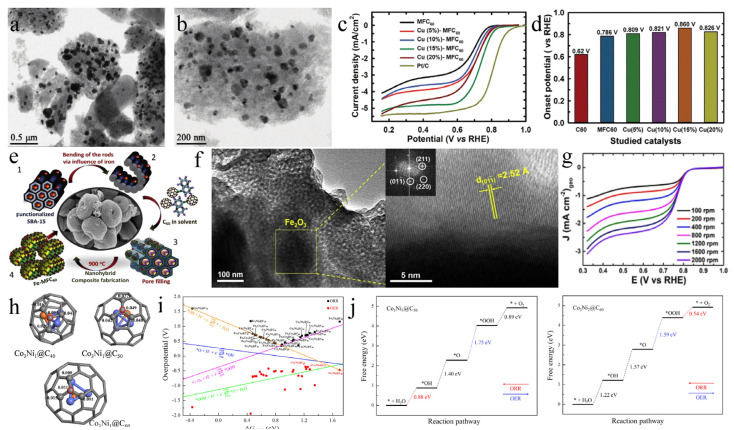
(**a**) Low and (**b**) high resolution TEM images of Cu(15%)-MFC_60_. (**c**) Consolidated ORR polarization curves were recorded at 1600 rpm in O_2_-saturated 0.1 M KOH (scan rate: 10 mV s^−1^) for the studied catalysts. (**d**) The respective onset potential. Reproduced with permission [61]. Copyright 2020, Elsevier Ltd. (**e**) Pictorial representation of mesoporous iron oxide C_60_ (Fe-MFC_60_). (**f**) TEM images of Fe-MFC_60_-150. (**g**) Linear Sweep Voltammetry (LSV) curves of Fe-MFC_60_-150 were recorded in O_2_-saturated 0.5 M KOH at different rotation speeds. Reproduced with permission [62]. Copyright 2019, Elsevier Inc. (**h**) Charge distributions on Co_3_Ni_1_@C_40_, Co_3_Ni_1_@C_50_, and Co_3_Ni_1_@C_60_. (**i**) The volcano relationship between overpotential and ∆G_*OH_. (**j**) Free energy diagrams of ORR and OER on Co_1_Ni_3_@C_50_ and Co_2_Ni_2_@C_60_. Reproduced with permission [66]. Copyright 2021, Elsevier B.V.

A catalyst’s support, which impacts the activity and durability of the catalyst [58], should have excellent electrical conductivity, corrosion resistance, and a large specific surface area, and ensure uniform and stable attachment of the active catalyst nanoparticles. C_60_ has the characteristics of a particular shape, strong donor-acceptor charge transfer ability, and easy-to-regulate morphology, which provide the possibility for it to become an excellent catalyst carrier [59,60]. Considering these factors, Gopalan Saianand et al. [61] prepared Cu/Cu_2_O nanoparticles (NPs) anchored on mesoporous fullerenes (MFC_60_) by hard template synthesis method and wet impregnation (Figure 5a,b). The obtained Cu/Cu_2_O-MFC_60_ catalysts with a 15 wt.% Cu/Cu_2_O NPs loading had the highest ORR catalytic activity among the investigated electrodes. It achieved an onset potential of 0.86 V vs. reversible hydrogen electrode (RHE) and a diffusion-limiting current density of −5.18 mA cm^−2^ (Figure 5c,d). In detail, the excellent catalytic activity of Cu/Cu_2_O-MFC_60_ was mainly attributed to the well-ordered mesoporous properties, abundant active sites, suitable specific surface area, and synergistic coupling effect of Cu/Cu_2_O NPs and C_60_. Mercy R. Benzigar et al. [62] adopted a hard template method to load highly crystalline α-Fe_2_O_3_ onto mesoporous C_60_ to synthesize a Fe-MFC_60_ catalyst (Figure 5e), which displays high ORR catalytic activity with an onset potential at 0.85 V vs. RHE and half-wave potential at 0.78 V vs. RHE (Figure 5f,g).

Studies have shown that metal encapsulation of carbon materials can also improve catalytic activity and stability [63,64]. Compared with other two-dimensional supported materials, the most significant difference of C_60_ is its larger hollow spherical structure, which allows encapsulating metal nanoparticles. He et al. [65] reported that M@C_60_ (M = Na, K, Rb, Cs, Sc, Ti, Mn, Fe) had a high HER catalytic activity when C_60_ was separated into 20 metal atoms, which was mainly because the charge transfer of metal atoms to C_60_ changes the charge distribution and enhances the adsorption strength of H atoms on M@C_60_. Chen et al. [66] focused on the catalytic performance of C_60_ encapsulated bimetals M1_x_M2_4−x_@C_n_ (M1_x_M2_4−x_ represents Fe_x_Co_4−x_, Fe_x_Ni_4−x_, Co_x_Ni_4−x_; x = 1, 2, 3; *n* = 40, 50, 60) by using DFT methods. Notably, the smaller fullerenes led to the greater charge transfer between the alloy core and the carbon shell, which was also confirmed by the most positive charges on the active site of Co_3_Ni_1_@C_40_ (Figure 5h). Furthermore, the volcano relationship indicated that Co_1_Ni_3_@C_50_ and Co_2_Ni_2_@C_60_ yielded high ORR activity (η^ORR^ = 0.35 V) and OER activity (η^OER^ = 0.36 V), respectively (Figure 5i,j).

#### 4.2.2. Molybdenum Disulfide

As the most representative transition metal dichalcogenide material, molybdenum disulfide (MoS_2_) is an excellent HER electrocatalyst due to its great planar active sites (active edges, S-vacancies, and grain boundaries) and high planar carrier mobility [67,68]. Yun-Hyuk Choi et al. [69] utilized a step-wise synthesis method including vapor transport, reduction, and topochemical sulfidation to grow 3D MoS_2_ nanosheets on carbon fiber paper (CFP) substrates. Then, they used a simple solution deposition method to prepare 3D MoS_2_ nanosheets and fullerene nanoclusters composite nC_60_/MoS_2_. The HER activity of nC_60_/MoS_2_ was significantly enhanced due to the improved interfacial charge transfer of the hybrid nC_60_/MoS_2_ p-n heterojunction. Based on the one-pot synthesis of vdW MoS_2_/C_60_ heterojunctions, Alain R. Puente Santiago et al. [70] studied the effect of C_60_ concentration on the HER catalytic activity. The results showed a solid interfacial interaction between C_60_ and MoS_2_ in 1T-MOS_2_/C_60_ supplemented with 20 wt% C_60_. The optimal binding strength of H atoms at the active site resulted in a Pt-like initial potential and an ultra-low ΔG_H*_(−0.03 eV).

#### 4.2.3. Other Graphite Derivatives

**Figure 6:**
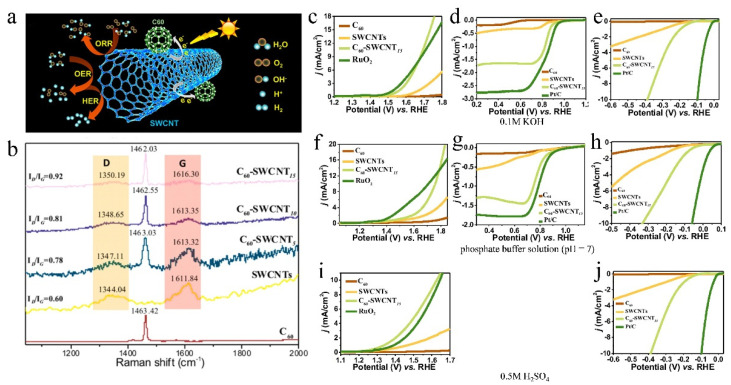
(**a**) Illustration of charge-transfer process and ORR/OER/HER on C_60_-SWCNTs. (**b**) Raman spectra of C_60_ and C_60_-SWCNT_n_ (*n* = 0, 5, 10, and 15 min). LSVs of (**c**) OER, (**d**) ORR, and (**e**) HER for pure C_60_, SWCNTs, C_6-_SWCNT_15_, and RuO_2_ in 0.1 M KOH. LSVs of (**f**) OER, (**g**) ORR, and (**h**) HER for pure C_60_, SWCNTs, C_60_-SWCNT_15_, and RuO_2_ in phosphate-buffered solution. LSVs of (**i**) OER, and (**j**) HER for pure C_60_, SWCNTs, C_60_-SWCNT_15_, and RuO_2_ in 0.5 M H_2_SO_4_. Reproduced with permission [73]. Copyright 2019, American Chemical Society.

C_60_ has become a key component of functional micro/nanostructures due to its unique spherical structure, excellent electron-accepting ability, and high electron conductivity [36,71]. Aliyeh Hasanzadeh et al. [72] synthesized C_60_-CNTs hybrid materials by covalently connecting fullerenes with carbon nanotubes for efficient ORR. C_60_-CNTs possessed a large specific surface area, good intermolecular electronic transitions, fast mass transport, and defective sp^3^-C bonds, which promoted O_2_ adsorption and OOH desorption. Gao et al. [73] reported a C_60_ as the electron acceptor adsorbed on SWCNTs, which effectively induced charge transfer between C_60_ and SWCNTs (Figure 6a). Raman spectra of C_60_-SWCNT_n_ (*n* = 5, 10, and 15 min) exhibit an upshift in the peak position with increasing C_60_ adsorption time (Figure 6b), which supports the charge transfer from SWCNTs to the electron-withdrawing C_60_. Moreover, the increased intensity ratio of the D-band to the G-band indicates that the nanotube structure became slightly more rich in defects (Figure 6b). The formed new metal-free, heteroatom/defect-free C_60_-SWCNTs material served as a multifunctional catalyst for ORR, OER, and HER over a wide pH range (Figure 6c–j).

## 5. Application of Carbon Nanotubes in Electrocatalysis

### 5.1. Doping Effect

#### 5.1.1. Nitrogen Doping

**Figure 7:**
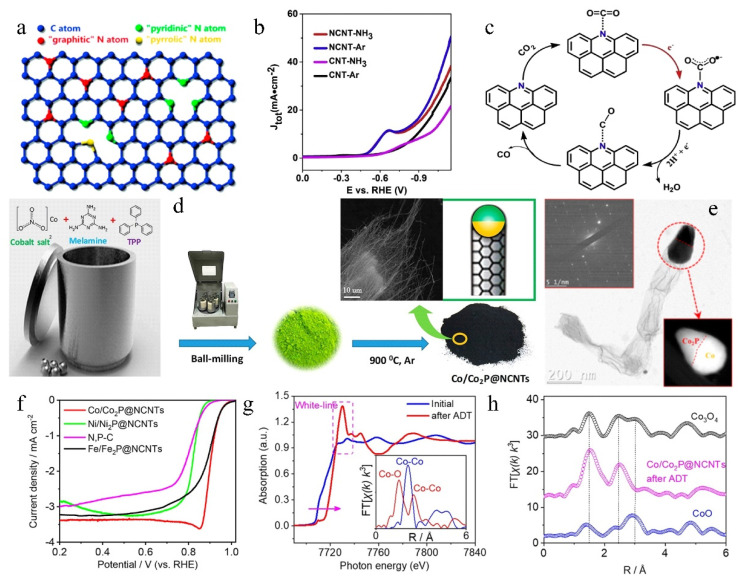
(**a**) Nitrogen doping into carbon plane at different locations. Reproduced with permission [75]. Copyright 2016, Elsevier Ltd. (**b**) LSV curves for NCNT-NH_3_, NCNT-Ar, CNT-NH_3_, and CNT-Ar in CO_2_-saturated 0.5 M NaHCO_3_ aqueous solution at 50 mV s^−1^. (**c**) Mechanism of the CO_2_ reduction reaction on NCNTs. Reproduced with permission [77]. Copyright 2019, American Chemical Society. (**d**) Schematic procedure for synthesizing the gram-scale Co/Co_2_P@NCNTs. (**e**) TEM images of the Co/Co_2_P@NCNTs. (**f**) ORR polarization plots of the M/M_2_P@NCNTs (M = Co, Ni, or Fe) and the N, P-C (rotation rate of 1600 rpm) in an O_2_-saturated 1.0 M KOH solution. (**g**) Normalized Co K-edge XANES spectra of the Co/Co_2_P@NCNTs before and after the accelerated cycling durability test, and (**h**) the corresponding magnitude Fourier transforms of Co K-edge EXAFS oscillations k^3^χ(k) (k weight of 3). The dashed vertical lines show the Co^3+^-O, Co^3+^ Oh-Co^3+^ Oh, and Co^2+^ Td-Co^3+^ Oh distances of 1.48, 2.44, and 3.03 Å, respectively. Reproduced with permission [83]. Copyright 2021, American Chemical Society.

Carbon nanotubes have been widely used in electrocatalysis due to their sufficient surface area, high conductivity, and well-established surface modification. However, the defects of CNTs, such as aggregation, chemical inertness, and solubility, are not insignificant. Existing methods, such as introducing defects, hetero-doping, and surface modification, have been used to modify CNTs. Notably, N-doping of carbon nanotubes (NCNTs)’ surfaces effectively controls the electronic structure and charge density distribution and generates more active sites, thus improving chemical reactivity [74]. Several locations accepting nitrogen doping in the carbon structure affect the catalytic activity of nitrogen-doped carbon materials. The common N doping types mainly include pyridinic N (398.6 EV), pyrrolic N (400.6 EV), and graphitic N (401.6 EV) (Figure 7a) [75]. Huang et al. [76] successfully developed NCNTs with fixed defect concentration by low-temperature preheating. They found that the higher the temperature for nitrogen doping, the higher the graphite N content. Moreover, combined with characterization, pyridinic N and pyrrolic N were identified as the active sites for the two-electron ORR pathway, while graphitic N accelerated the four-electron ORR pathway. The precise nitrogen doping can not only determine the real active sites of the catalyst but also explore the relationship between structure and properties. Ma et al. [77] synthesized NCNTs with a high concentration of pyridinic N (62.3% of the total nitrogen) by pyrolysis. The high concentration of pyridinic N, combined with gas-phase CO_2_ electrolysis, was proved to effectively enhance the enrichment of CO_2_ on the surface of NCNTs, which promoted the subsequent CO_2_RR reaction (Figure 7b). Furthermore, based on the DFT calculations of the CO_2_ reduction reaction on NCNTs, the author demonstrated that electron transfer (red arrow in Figure 7c) becomes the rate-determining step (RDS), which attributes to high pyridinic N concentration.

Nitrogen-doped carbon nanotubes’ wall number and growth mode also affect catalytic activity. Zhang et al. [78] found that NCNTs with an average wall number of 2.5 had higher ORR catalytic activity. For this sample, the inner layer provided an effective conductive path to transfer electrons from the inner layer to the outer layer through the tunneling effect. Nevertheless, the tunneling effect became weaker with increasing or decreasing the wall number of NCNTs, leading to the falling catalytic activity of NCNTs, which was further experimentally validated by others [79,80]. Besides, Yang et al. [81] focused on the active source of pure SWCNTs and non-metal-doped SWCNTs in the ORR process. They found that the pyramidalization angle is an excellent descriptor to study ORR activity on nitrogen- and boron-doped and undoped SWCNTs through machine learning tools, which enables prediction of the optimal diameter and the best doping type for the SWCNTs surfaces during the ORR. Li et al. [82] successfully prepared cactus-like NCNTs by directional growth using layered double hydroxides (LDHs) as catalyst precursors and metal-organic frameworks (MOFs) particles as carbon and nitrogen sources. Due to the unique hierarchical array structure, uniform N doping, and low charge transfer resistance, NCNTs yielded high catalytic activity in ORR and OER. Wu et al. [83] developed Co/Co_2_P@NCNTs catalysts with Co/Co_2_P heterojunction encapsulated in bamboo-like N-doped carbon nanotubes (Figure 7d,e). The Co/Co_2_P@NCNTs with the effect of abundant pyridinic N and graphitic N active sites, and highly ordered NCNTs, significantly enhanced the ORR kinetics and effectively attenuated the negative effects of high oxidation potential (during the OER process) on the ORR performance in alkaline electrolyte, showing high ORR activity with a half-wave potential (E_1/2_) of 0.87 V (Figure 7f). Meanwhile, the dynamic active state transformation from the Co/Co_2_P heterojunctions into Co^3+^ Oh-containing CoO_x_(OH)_y_ active species contributed to the markedly improved OER catalytic activity (Figure 7g,h).

#### 5.1.2. Polyatomic Doping

Carbon nanotubes doped with two or more heteroatoms can also significantly improve the catalytic performance, owing to the existence of doped atoms and the effect of synergistic coupling on carbon nanotubes. Qu et al. [84] synthesized N,S co-doped carbon nanotubes (N,S-CNT) by the two-step “graft-and-pyrolyze” method. The N,S-CNT catalysts with uniform and high concentration of S doping (5.6 at%) displayed superb OER and HER bifunctional catalytic activities in alkaline electrolytes. Furthermore, secondary S-doping had a crucial role in forming electrocatalytically active sites and enhancing charge transfer. Liu et al. [85] demonstrated that Ru@Co/N-CNTs were highly functional for HER in acid and alkaline electrolytes by anchoring Ru nanoclusters on Co/N-doped carbon nanotubes. In detail, the as-prepared optimal catalyst showed a remarkable performance with low overpotentials of 48 and 92 mV at 10 mA cm^−2^ in alkaline and acidic media, respectively. The excellent stability and hydrogen production efficiency of Ru@Co/N-CNTs were mainly attributed to a large ECSA and high exposure of Ru active sites. Based on the successful synthesis of B, N co-doped graphene nanosheets (BCN), Hassina Tabassum et al. [86] used polyethylene glycol (PEG) with different molecular weights as guiding agents to roll BCN into BCN nanotubes with adjustable sizes and atomic bonds. The synthetic catalyst with a large specific surface area, abundant active sites, high concentration of pyridinic N, and numerous B-C, N-C bonds exhibited high ORR and HER bifunctional catalytic activity.

### 5.2. Carrier Effect

Carbon nanotubes can be composited with monoatomic metals [87], metal oxides [88,89,90], and other graphite-derived carbon materials [91], to improve activity and durability [92]. As supported catalysts, it not only acts as a conductive carrier but also controls the electron distribution on the surface by utilizing the interaction with the supporting materials.

**Figure 8:**
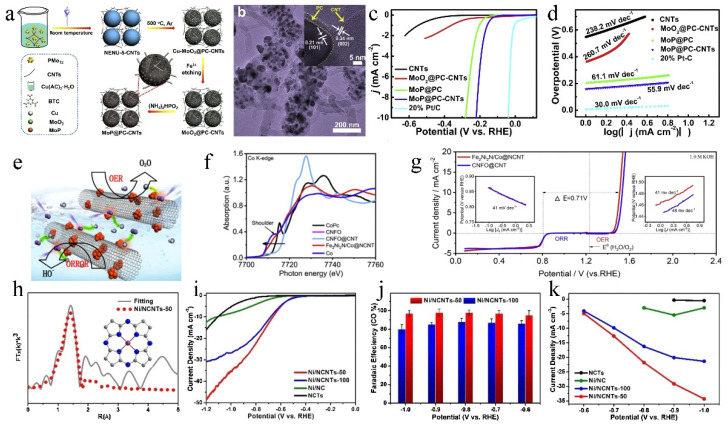
(**a**) Schematic illustration of the fabrication of MoP@PC-CNTs nanocomposite. (**b**) TEM images of MoP@PC-CNTs. (**c**) HER LSV curves of different catalysts and (**d**) the corresponding HER Tafel plots. Reproduced with permission [93]. Copyright 2018, Elsevier Ltd. (**e**) Illustration of ORR and OER on Fe_2_Ni_2_N/Co@NCNT. (**f**) Co K-edge XANES and spectra of various catalysts and standard samples, including FePc (Iron (II) phthalocyanine), CoPc (Cobalt (II) phthalocyanine) and NiPc (Nickle (II) phthalocyanine). (**g**) LSV curves for ORR and OER of Fe_2_Ni_2_N/Co@NCNT in O_2_-saturated 1.0 M KOH at a scan rate of 5 mV s^−1^, with the inset showing ORR (**left**) and OER (**right**) Tafel plots of the Fe_2_Ni_2_N/Co@NCNT. Reproduced with permission [94]. Copyright 2019, Elsevier Ltd. (**h**) The corresponding EXAFS fitting curves of Ni/NCTs-50. (**i**) LSV curves, (**j**) FE^CO^ and (**k**) CO partial current densities for Ni/NCTs-50, Ni/NCTs-100, Ni/NC and NCTs in CO_2_-saturated 0.5 M KHCO_3_ solution at various applied potentials. Reproduced with permission [95]. Copyright 2020, Elsevier B.V.

The beneficial effect of carbon nanotubes as a support has been demonstrated by strengthening the in-plane support and electrical conductivity of the composites. Li et al. [93] first synthesized pomegranate-like MoP@PC-CNTs by simple carbonization and phosphating process with POMOFs-CNTs composite as a precursor (Figure 8a,b). The introduction of CNTs offered more catalytic sites and enhanced long-range conductivity. Benefiting from the carrier, the composite displayed a low onset overpotential of 75 mV and a small Tafel slope of 55.9 mV dec^−1^ for the HER (Figure 8c,d). Wu et al. [94] prepared an ORR and OER bifunctional electrocatalyst (Fe_2_Ni_2_N/Co@NCNT) with nanoclusters uniformly anchored on nitrogen-doped carbon nanotubes (Figure 8e,g). Due to the coupling effects in Fe_2_Ni_2_N/Co@NCNT, the electron transfer from the metal atoms (Fe, Ni) to the neighboring N and O atoms was revealed by the analysis of XAFS (Figure 8f). At the same time, the NCNT accelerated exchange kinetics of O^2−^/OH^−^ and provided abundant contact area, strong adhesion, and low aggregation of Fe_2_Ni_2_N/Co nanoclusters. Hou et al. [95] preparedcore–shell nanorods by coating ZnO with bimetallic zeolitic-imidazolate framework-NiZn (ZIF-NiZn), to obtain porous N-doped carbon nanotubes stabilized Ni SACs (Ni/NCTs) by a pyrolysis process. Based on EXAFS curves, the fitting result showed that the coordination number of Ni-N in Ni/NCTs-50 is near to that of NiPc with Ni-N_4_ structure (Figure 8h). Due to the porous nanotube structure, high specific surface area, and atomized Ni-N coordination active sites, Ni/NCTs exhibited superior CO_2_RR activity with a CO Faradaic efficiency of nearly 100% over a wide potential range of −0.6 V to −1.0 V vs. RHE (Figure 8i–k).

## 6. Application of Graphene in the Field of Electrocatalysis

### 6.1. Heteroatom Doping

Due to the advantages of extremely high surface area, high electron mobility, and variations of graphene doping structures, graphene has broad application prospects in various fields [96]. However, the inert carbon plane and zero band gap structure of impurity-free graphene, which exhibits poor electrocatalytic activity, are unsuitable for electrocatalysis [97]. Considering graphene’s inert structure, heteroatom doping becomes an essential approach in graphene modification [98]. Graphene doping elements mainly include N, P, B, and S, which introduce defects, change the electronic structure near the doped graphene atoms, and introduce more active sites, thus improving the catalytic activity [99,100,101].

#### 6.1.1. Single Atom Doping

N-doping has been the most intensively studied in the graphene doping electrocatalytic material. The role of N-doped graphene’s active sites in ORR catalysis is still controversial. Yan et al. [102] performed simulation calculations on pure graphene, graphitic N, pyridine N, and graphene doped with graphitic N and pyridine N, respectively. The result indicated that the composite doping of graphitic nitrogen and pyridine nitrogen achieves charge redistribution, thereby promoting the adsorption of O_2_. Compared to mono-N-doping in graphene, binary-N-doped graphene possessed excellent catalytic activity for the CO_2_RR due to its stable adsorption of reactants [103]. Wang et al. [104] introduced several disordered structures through high-concentration KOH etching based on N-doped graphene. The experiments reveal that the high HER activity came from more active sites of dual defective graphene-based materials. In addition, many studies demonstrate that S- P-doping, B- P-doping, and P-doping also enhance the catalytic performance of graphene [105,106,107,108]. For instance, Li et al. [109] employed DFT to explore the ORR activity and mechanism of heteroatom-doped graphene catalysts with single X-doped graphene (X = N, P, As, Sb, S). They find that binding energies of *OH (ORR intermediates) on the catalysts can serve as a descriptor for the ORR activity, which was attributed to the abundance of electronic states at the Fermi level.

#### 6.1.2. Polyatomic Doping

Compared with single heteroatom doping, polyatomic co-doped graphene is easier to introduce defects and modification of the electronic structure due to the synergistic effect between doping atoms, consequently leading to the enhancement of electrocatalytic activity [110,111,112]. Liang et al. [113] selected highly active N and S atoms as dopants to prepare N, S double-doped graphene (N-S-G). The ORR performance of N-S-G was significantly better than that of S single-doped(S-G) or N single-doped (N-G) catalysts. Additionally, DFT calculation confirmed that N, S double-doped graphene resulted in the redistribution of spin and charge density, leading to the enhancement of synergistic catalytic activity. Among double-doped graphene, the incorporation of metal elements enhances the electrical conductivity of doped graphene, thus exhibiting efficient catalytic performance [114,115,116]. Furthermore, Zhang et al. [117] prepared N, P, and F tri-doped graphene by a pyrolysis method. The corresponding synergistic effect of the doping atoms created highly active graphene-based ORR, OER, and HER catalysts.

### 6.2. Graphene Supported Metal

Because of the high electron transfer [6], advanced pore structure [118], great specific surface area [119], and easy coupling and synergistic effect with metals [120,121], graphene, especially doped graphene, has become a very popular candidate as a metal catalyst carrier.

#### 6.2.1. Single Atom Catalysts

**Figure 9:**
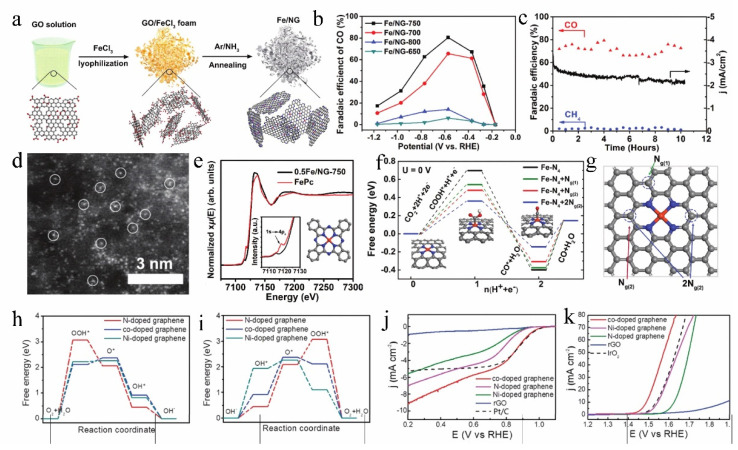
(**a**) Schematic of the synthesis process of the Fe/NG catalyst. (**b**) Potential dependence of CO FE for electrochemical CO_2_ reduction on Fe/NG catalysts prepared at different annealing temperatures (in aqueous 0.1 m KHCO_3_). (**c**) Chronoamperometric curves of stability test with Fe/NG-750 at −0.60 V versus RHE in the CO_2_-saturated 0.1 M KHCO_3_ solution. (**d**) HAADF-STEM images of Fe/NG−750 catalyst. (**e**) Normalized Fe K edge XANES spectra of 0.5Fe/NG-750 catalyst (black line) and FePc reference (red line); the inset shows the enlarged view of pre-edge features. (**f**) Free energy diagram for electrochemical CO_2_ reduction to CO on FeN_4_ moieties embedded on graphene sheets. (**g**) Top view of the optimized structures for Fe-N_4_ moieties embedded on graphene layer and potential nitrogen-substitution. Reproduced with permission [125]. Copyright 2018, Wiley-VCH. Free energy diagrams for (**h**) the ORR and (**i**) OER processes. (**j**) ORR polarization curves of different electrodes in O_2_-saturated 0.1 M KOH. (**k**) OER polarization curves of different electrodes in 1.0 m KOH. Reproduced with permission [129]. Copyright 2019, Wiley-VCH.

Single-atom catalysts (SACs) have attracted extensive attention due to their sufficient atomic efficiency, high catalytic activity, and excellent selectivity among electrocatalytic materials. However, single-atom agglomeration without substrate has dramatically impeded the limited performance [122]. Doped graphene effectively alleviates atom agglomeration and provides a fantastic conductive substrate, enriches loading sites, and enhances single atom adsorption [123,124]. Zhang et al. [125] employed graphene oxide (GO) as a precursor to anchor atomic Fe-N_4_ to nitrogen-doped graphene (Fe/NG) through simple heat treatment, yielding a catalyst with better CO_2_RR catalytic activity, high selectivity, and stability (Figure 9a–c). The isolated Fe-N_4_ structure is more critical for the reduction of CO_2_ to CO, which was confirmed by XAFS (Figure 9d,e). Furthermore, the mechanism of the CO_2_ reduction reaction on Fe-N_4_ moieties embedded in N-doped graphene showed a potential promotional effect of nitrogen-doping of graphene (Figure 9f,g). Li et al. [126] also demonstrated that Fe-N_4_ has impressive activity for CO_2_RR. Nitrogen-doped graphene-supported single Mo atoms (Mo@NG) [127] and single Ni atoms (Ni-NG) [128] have been confirmed to improve the CO_2_RR catalytic activity. However, the active sites of Mo@NG and Ni-NG were not the M-N_4_ structure but the high dispersion of single metal atoms, abundant atomic catalytic efficiency, and the combined metal-N effect. Besides, N-doped graphene-supported single-atom Ni also exhibited unusual OER and ORR activities, which were attributed to the rich Ni doping, porous structure of N-doped graphene, and Ni, N co-doping (Figure 9h–k) [129].

#### 6.2.2. Metals and Metal Oxides

Apart from highly dispersed metal single atoms, metal nanoparticles or metal nanoclusters also have particular activity owing to stable geometric structures, metal strain effect, and lattice defects [125,130,131,132]. Wang et al. [133] reported an electrocatalyst (Ir-NSG) with uniformly dispersed and intercalated Ir nanoclusters into N, S co-doped graphene. The superb performance in HER and OER originated from the Ir site’s electronic state and coordination environment. N and S doping optimized the adsorption of hydrogen and oxygen intermediates on the Ir site and accelerated both HER and OER reaction kinetics. Meanwhile, N, S doped graphene provided a durable carrier and sufficient adsorption sites for Ir nanoclusters. Huang et al. [134] focused on the combination of metal nanoparticles and graphene, producing dispersed oxidized cobalt nanoparticles (5 nm) onto the monolayer of single-layer nitrogen-doped graphene (PO-5 nm Co/SL-NG) by a simple one-pot synthesis strategy. The synergistic effect of proton and electron multiple transfers in the CO_2_RR process is attributed to the high surface area, high conductivity, and synergy with PO-5 nm Co of SL-NG. Besides the role of catalyst support, graphene can also act as a protective layer by coating metal nanoparticles [135]. For instance, a single-layer graphene covering the Cu surface effectively weakened the morphological changes of Cu during the electrocatalysis process and improved the catalytic stability [136]. In addition, metal oxides also yield good catalytic efficiency and selectivity due to the oxygen vacancy defects caused by oxygen introduction [137,138,139,140]. Particularly, Zhang et al. [141] deposited ultrasmall SnO_2_ nanocrystals on the surface of nitrogen-doped graphene (SnO_2_/rGO) via an in situ conversion strategy, which resulted in an enhancement of the conversion efficiency and selectivity in the CO_2_RR. The oxygen vacancies in SnO_2_ nanocrystals minimized severe agglomeration and poor electrical conductivity.

#### 6.2.3. Other Metal Compounds

Benefits arising from the changes in the electronic structure and coordination environment caused by non-metallic elements, metal phosphides [142], metal nitrides [143], and metal sulfides [144] have tremendous implications for electrocatalysis applications [145,146]. Guo et al. [147] synthesized (N, S)-RGO@CoN by combining spray drying and atomic layer deposition, producing a catalyst that showed efficient and durable OER performance in the neutral electrolyte. The improved OER performance was related to the synergistic effects of short charge transfer paths, abundant active sites, and stable chemical coupling with CoN provided by the (N, S)-RGO substrate. At the same time, the unique 3D structure of P, S double-doped rGO(PSG) had also been confirmed to be beneficial for exposing more active sites and promoting the mass transfer of the electrolyte to electroactive sites on the electrocatalyst [148].

### 6.3. Graphene Quantum Dots

**Figure 10:**
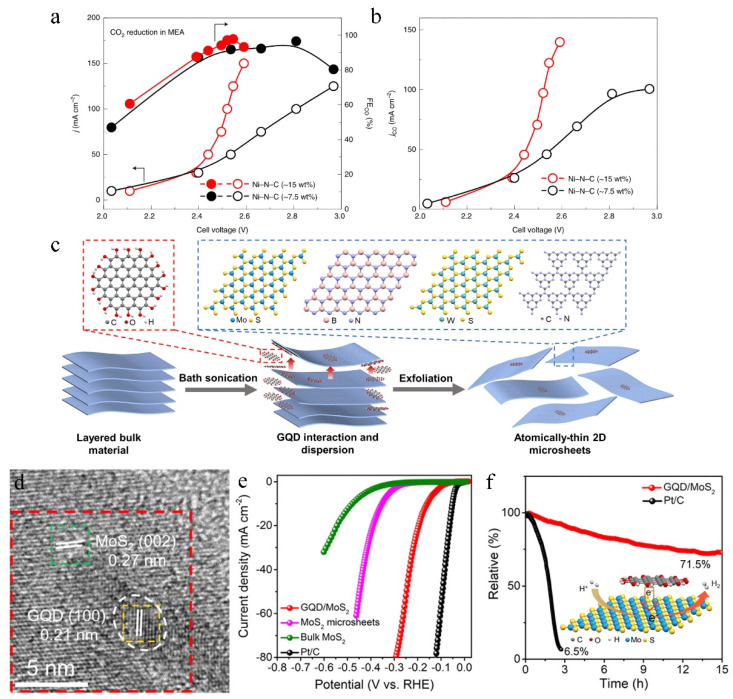
(**a**) The steady-state current densities and the corresponding Faradaic efficiencies of CO (FE_CO_) of ~7.5 wt% Ni-N-C and ~15 wt% Ni-N-C catalyst in an anion membrane electrode assembly (MEA). (**b**) The corresponding CO partial current densities (j_CO_) of ~7.5 wt% Ni-N-C and ~15 wt% Ni-N-C catalyst at different applied cell voltages. Reproduced with permission [153]. Copyright 2021, Nature Publishing Group. (**c**) Schematic illustration of GQD-assisted exfoliation of MoS_2_, h-BN, WS_2_ and g-C_3_N_4_ microsheets. (**d**) HRTEM images of GQD/MoS_2_. (**e**) Polarization curves of bulk MoS_2_. (**f**) Stability test of GQD/MoS_2_ and Pt/C. Reproduced with permission [158]. Copyright 2021, Elsevier Ltd.

As the carbon material family’s new member, graphene quantum dots (GQDs) are a 0D graphene material, which is characterized by 1 or 2 layers of graphitic planes with lateral dimensions typically <10 nm [149]. Compared with 2D graphene, the GQDs are currently explored as potential electrocatalysis due to unique advantages such as excellent dispersion, high surface area, facile chemical modification, abundant active sites, and surface functional groups [150,151,152]. When the size of the carrier is reduced to the GQDs level, the single atom on the catalyst surface is isolated from each other, which can impressively improve that single atomic load. For example, Xia et al. [153] used GQDs as intermediate carbon supports to increase the loading of Ni atoms, thereby improving the catalytic activity of the CO_2_RR reaction (Figure 10a,b). Simultaneously, Tran Van Tam et al. [154] focused on doped graphene quantum dots (BGQDs) with higher B doping content (4.25%), which improved CO_2_RR catalytic activity compared to GQDs. Compared with N single-doped GQDs, the N and S Co-doped GQDs changed the N doping state due to the introduction of S, resulting in the generation of asymmetric spin and the increase of charge density, thus showing improved activity [155]. In addition, heterojunctions have become an emerging frontier trend in electrocatalysis due to their synergistic effects, strain effects, and electronic interactions [156]. Gong et al. [157] reported a strategy to compound 2D microsheets with a large number of 0D/2D van der Waals heterojunctions (vdWHs) on the surface (Figure 10c,d). Using amphiphilic GQDs as intercalators and dispersants, the N and S Co-doped GQDs formed van der Waals heterojunctions with 2D graphene sheets. The GQD/MoS_2_ van der Waals heterojunctions(GQD/MoS_2_ vdWHs) significantly reduced HER overpotential and improved the electrode’s long-term stability because of the synergistic coupling effect with the OD/2D heterojunction (Figure 10e,f) [158].

### 6.4. Other Graphene-Based Composites

Given the strong van der Waals interactions in the preparation process, graphene is prone to aggregation and stacking, which reduces active sites and mass transport rate during the catalytic process, which seriously affects its electrocatalytic activity [159,160,161]. To solve these issues, several feasible methods have been reported for designing 3D structure nanocomposites composed of carbon nanotubes [162], 3D graphite foams [163,164], and graphene. Yang et al. [165] assembled carbon nanotubes and graphene into N, P co-doped hybrid nanosphere aerosols (N, P-CGHNs), which effectively prevented graphene stacking. The hybrid structure could form efficient charge transfer pathways that synergistically improved the ORR reaction electron transfer efficiency. Moreover, Mohammad Tavakkoli et al. [166] prepared N-Co-Mo-GF/CNT loaded simultaneously with single atoms of N, Co, and Mo by vapor deposition method using the graphene nanosheet (GF)-carbon nanotube (CNT) hybrid structure as the carrier. This GF/CNT, with high specific surface area and mesoporous structure, promoted mass transport during the catalytic reaction, thereby enhancing the catalytic activity of the ORR and OER reactions. However, in contrast to other hybrid designs of graphene and carbon nanotubes, Lai et al. [167] constructed N, S co-doped carbon nanotube/graphene nanosheet composites (N−S−CNTs) with a unique 3D structure, yielding high electrical conductivity, uniform dispersion of Ni_3_Fe, and exposure of active electrocatalytic sites.

## 7. Conclusions and Perspective

The recent advances in the design of electrocatalysts for ORR, OER, HER, and CO_2_RR based on graphite-derived materials have been summarized in this manuscript, and the performance-related information is presented in Table 1. There are generally two strategies to improve the electrocatalytic activity: (i) increasing the intrinsic activity of catalysts, and (ii) increasing the number of exposed active sites. This paper discusses structural regulation strategies and carrier function of graphite-derived materials for the above-mentioned electrochemical reactions in terms of: (1) hetero doping modification, (2) defect control, (3) heterojunction introduction, and (4) uniformity of metal active electrocatalyst dispersion.

Based on the review of the literature presented in the current manuscript, the following are the recommendations proposed:

(1) Deeper insights into the electrocatalytic active sites of modified graphite-derives are required, especially doped graphite-derived materials. Advanced operando characterization methods are also necessary to deeply explore the effect of doping on the electronic distribution of active sites. By combining theoretical DFT simulations and various advanced in situ characterization methods, including in situ X-ray diffraction (XRD), X-ray absorption spectra (XAS), Raman, and Fourier-transform infrared (FTIR), the role of doping can be well understood.
(2) Systematic understanding of the carrier role of graphite-derived materials. Due to the large specific surface area, easily regulated structures, and abundant active sites, the fullerenes, carbon nanotubes, and graphene can act as active catalysts and catalyst support for other active materials. Furthermore, the interfacial behavior between the carrier and the active catalyst should be paid more attention.
(3) Catalyst activity measurement standards should be established to facilitate the comparison of the activity of electrocatalysts. Although researchers have developed many electrocatalysts over a few decades, it is still challenging to compare their performances due to the nonstandardized measurements (see Table 1). Therefore, the reports must establish a standard to appropriately and accurately compare electrocatalysts for ORR, OER, HER, and CO_2_RR.

## Figures and Tables

**Table 1:** Summary of Catalytic performance of discussed catalysts in the main text.

Catalysts	Strategies	Catalytic Performance/vs. RHE	Ref.
Cu/Cu_2_O-MFC_60_	Loaded Defect	ORR 0.86 V@E_onset_, −5.183 mA cm^−2^@diffusion-limiting current density	[61]
Fe-MFC_60_	Doped Loaded	ORR 0.85 V@E_onset_, 0.78 V@E_1/2_	[62]
ANG	co-doped	ORR 0.99 V@E_onset_, 0.85 V@E_1/2_, 4.5 mA cm^−2^ Current density at 0.8 V	[114]
CPS@G_N,S,P_	Doped Loaded	ORR 0.8 V@E_1/2_, 29 mV dec^−1^@ Tafel slope	[142]
N,P-CGHNs	Doped Loaded	ORR 0.94 V@E_onset_, 0.82 V@E_1/2_	[165]
10% F/BCN	Doped Loaded	ORR 0.92 V@E_onset_, 0.79 V@E_1/2_, 12 h at 0.75 V@ Stability OER 390 mV@η10, 79 mV dec^−1^@ Tafel slope HER 0.042 V@E_onset_, 87 mV dec^−1^@ Tafel slope	[71]
Co/Co_2_P@NCNTs	Doped Loaded	ORR 0.90 V@E_1/2_ OER 480 mV@η50, 1.58 V@E_j=10_	[83]
np-graphene	co-doped Defect	ORR 96% current retention after a long-term 50 h test OER 1.45 V@E_onset_, 270 mV@η10, 59 mV dec^−1^@ Tafel slope	[129]
S-Ni_3_FeN/NSG	co-doped	ORR 0.878 V@E_1/2_, 40 mV dec^−1^@ Tafel slope OER 260 mV@η10, 76 mV dec^−1^@ Tafel slope	[143]
Ni_3_Fe/N-S-CNTs	co-doped Loaded	ORR 0.877 V@E_1/2_, 353 mV@η10, 43.2 mV dec^−1^@ Tafel slope OER 215 mV@η10, 44.1 mV dec^−1^@ Tafel slope	[167]
N,S-CNT	co-doped	OER 1.59 V@E_j=10_, 56 mV dec^−1^@ Tafel slope HER −0.4 V at 5 mA cm^−2^, 133 mV dec^−1^@ Tafel slope	[84]
Co_2_P@N,P-PCN/CNTs	co-doped Loaded	OER 280 mV@η10, 72 mV dec^−1^@ Tafel slope HER 154 mV@η10, 52 mV dec^−1^@ Tafel slope	[88]
Ir-NSG	co-doped Loaded	OER 307 mV@η10, 74.2 mV dec^−1^@ Tafel slope HER 22 mV@η10, 21.2 mV dec^−1^@ Tafel slope	[133]
Ru@Co/N-CNTs	co-doped Loaded	HER in 1 M KOH 48 mV@η10, 33 mV dec^−1^@ Tafel slope, 0.25 s^−1^ at -0.05 V @ TOF HER in 0.5 M H_2_SO_4_ 92 mV@η10, 45 mV dec^−1^@ Tafel slope	[85]
R-PtO_x_/CNT	Doped Loaded	HER 19.4 mV@η10, 34.6 mV dec^−1^@ Tafel slope	[89]
Ni/NiS/P,N,S-rGO	co-doped Defect	HER 155 mV@η10, 135 mV dec^−1^@ Tafel slope	[144]
GQD/MoS_2_	van der Waals heterojunction	HER 160 mV@η10, 56.9 mV dec^−1^@ Tafel slope	[158]
NCNTs	Rich-doped	CO_2_RR > 94.5%@FE, 20.2 mA cm^−2^at -(0.6–0.9 V)	[77]
Ni/NCTs-50	Doped Loaded	CO_2_RR 9366 h^−1^@TOF, 98%@FE, 34.3 mA cm^−2^ at −1.0 V	[95]
Fe/NG-750	Doped Loaded	CO_2_RR ≈ 80%@FE	[125]
PO-5 nm -Co/SL-NG	Doped Loaded	CO_2_RR(versus SCE) 380 mVη10, 71.4%@FE at −0.90 V	[134]
SnO_2_@N-rGO	Doped Loaded	CO_2_RR 21.3 mA cm^−2^at −0.8 V, 89%@FE	[141]
15 wt% Ni-N-C	GQD loaded high single-atom	CO_2_RR 122 mA cm^−2^ @CO partial current	[153]

## Data Availability

Not applicable.
